# Periorbital Silicone Granulomatosis 30 Years after Acupuncture

**DOI:** 10.1155/2020/6323646

**Published:** 2020-06-24

**Authors:** Nathan Pirakitikulr, Ann Q. Tran, Armando L. Garcia, Sander R. Dubovy, Wendy W. Lee

**Affiliations:** ^1^Division of Oculofacial Plastic and Reconstructive Surgery, Bascom Palmer Eye Institute, University of Miami-Miller School of Medicine, Miami, FL, USA; ^2^Florida Lions Ocular Pathology Laboratory, Bascom Palmer Eye Institute, University of Miami Miller School of Medicine, Miami, FL, USA

## Abstract

Silicone-based compounds are commonly used in many medical applications, such as coatings for needles and syringes. Foreign body granulomas are a well-recognized complication of silicone exposure; however, they may be challenging to identify without a clear history. A 61-year-old female patient without prior history of periocular injections, filler, or surgery presented to our oculoplastic clinic with multiple periocular lesions. The patient subsequently underwent excisional biopsy of two prominent lesions, which were identified as granulomas on pathology. Further questioning revealed the cause to be facial acupuncture performed decades prior, and a subsequent targeted exam identified additional lesions at other needling sites. A third lesion was subsequently excised, and there was no recurrence at the last follow-up 3 months postsurgery. Acupuncture is an increasingly common but underrecognized source of silicone exposure and can present up to several decades after exposure as a chronic granulomatous response in a characteristic multifocal pattern.

## 1. Introduction

Silicone-based compounds are commonly used in many medical applications, such as coatings for needles and syringes, as implants and prostheses, and as injectable cosmetic fillers. Foreign body granulomas have been recognized as a complication of silicone exposure since 1964 [1]. They may occur anywhere from months to decades after exposure [2]. Awareness of this risk has led to the decline of free silicone-based injectable fillers in favor of hyaluronic acid- and collagen-based fillers. In this report, we describe a patient without prior filler who presented to our oculoplastic clinic for evaluation of multiple raised periorbital nodules [3]. At the request of the patient, who was concerned the lesions that seemingly arose *de novo* could be malignant, an excisional biopsy was performed. Histopathological evaluation ultimately identified the lesions to be silicone granulomas. Further history revealed an uncommon exposure: facial acupuncture performed three decades prior. To our knowledge, this is the first reported case of multifocal periorbital silicone granulomas arising from prior acupuncture. Patient consent was obtained for publication of the medical photography included in the article. This report adhered to the ethical principles outlined in the Declaration of Helsinki as amended in 2013.

## 2. Case Presentation

A 61-year-old previously healthy Hispanic female presented for oculoplastic evaluation after noting multiple nontender bumps around the left eye associated with intermittent eyelid edema. The patient had no history of trauma and disclosed no prior facial surgeries at the initial visit. Past medical and social history was largely unremarkable. The patient was diagnosed with osteoporosis and was taking calcium supplementation. She reported high stress levels and was taking anxiolytics as needed. She had undergone LASIK several years prior for myopia. The patient did not smoke and did not endorse significant sun exposure.

**Figure 1:**
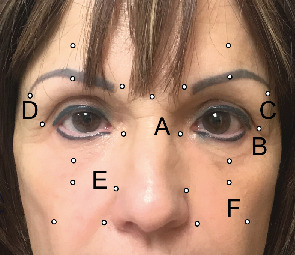
Acupuncture trigger points and sites of granuloma formation. Granulomas were found at sites labelled (a)–(f), which correspond to common acupuncture trigger points: (a) jingming, (b) tongziliao, (c, d) taiyang, (e) bitong, and (f) quanliao.

On exam, the patient was found to have multiple nontender, nonpigmented nodules without overlying skin changes at the following locations: below the left medial canthal tendon measuring 1 cm (Figure 1(a)), below the left lateral canthal tendon measuring 0.5 cm (Figure 1(b)), and lateral to the left eyebrow measuring 0.75 cm (Figure 1(c)). The patient subsequently underwent excisional biopsy of both canthal lesions (Figures 1(a) and 1(b)).

**Figure 2:**
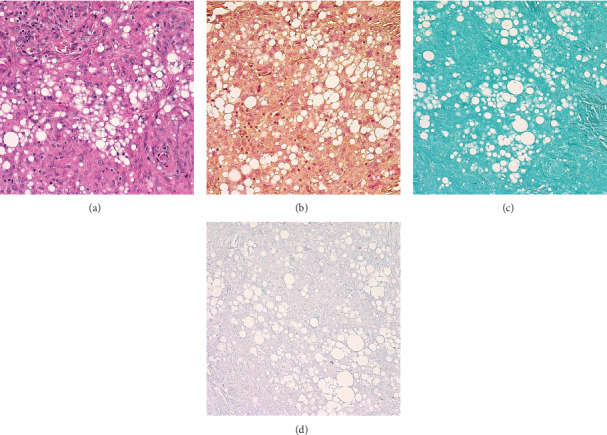
Histopathology of periorbital granuloma (400x magnification). (a) Hematoxylin and eosin stain demonstrate noncaseating granuloma with multiple foci of clear vacuole-like spaces containing silicone. No organisms were seen on (b) gram, (c) GMS, and (d) acid-fast stains.

Hematoxylin and eosin stain of the two excised specimens revealed fibrovascular tissue containing granulomatous inflammation with foci of clear spaces consistent with silicone oil or other foreign material (Figure 2(a)). No organisms were seen on gram stain, Grocott's methenamine silver stain, or acid-fast staining (Figures 2(b)–2(d)).

At follow-up, the patient disclosed a history of facial acupuncture performed approximately 30 years prior over multiple sessions at a local South Florida spa to relieve stress. The treatment was performed by an acupuncturist licensed by the Florida Board of Acupuncture using single-use disposable acupuncture needles. A comprehensive review of the patient's medical and surgical history revealed a previously undisclosed history of a minifacelift without fat transfer, Botox to the forehead, and permanent eyeliner tattooing. There were no records of other facial cosmetic procedures, and the patient denied any prior periocular lower lid injections or fillers. Given the additional exposure history, the patient was reexamined, and additional nodules along the right brow (Figure 1(d)), right nasolabial groove (Figure 1(e)), and left canine fossa (Figure 1(f)) were found, which corresponded to trigger points commonly used in acupuncture.

The patient underwent additional removal of the right brow lesion (Figure 1(d)), but elected to observe the deeper lesions found in the mid and lower face (Figures 1(e) and 1(f)).

## 3. Discussion

Despite the declining use of silicone-based fillers in cosmetic surgery, silicone granulomas are still frequently encountered by dermatologists and facial or oculoplastic surgeons. The diagnosis is generally made based on a clear history of exposure, such as through injectable fillers or implants, and characteristic histopathological findings [4]. In the presented case, the prior exposure was not initially disclosed until the histopathological findings prompted a guided review of the patient's history. It is important to ascertain any history of prior acupuncture as patients can be repeatedly exposed to the silicone used to coat needles. In this unique case, the patient presented with multiple periorbital silicone granulomas several decades after facial acupuncture.

Silicone granulomas following acupuncture have been previously described in several parts of the body and in some cases several years after exposure [5, 6], but have not been previously reported in the periocular region despite being a commonly needled site. To our knowledge, this is also the first report of periorbital silicone granulomas occurring decades after exposure. Due to the long span of time between exposure and the patient's clinical presentation, other diagnoses were considered, including cutaneous sarcoidosis. However, a first diagnosis of cutaneous sarcoidosis would have been rare in a 61-year-old patient [7, 8], and the patient had no clinical evidence of scarring at other sites of trauma or surgery. There were no pulmonary or systemic symptoms, and recent routine bloodwork performed by her primary care physician, which included serum calcium, was within normal range. Moreover, the histopathological findings pointed to prior silicone exposure. Notably, the abundance of vacuolated foreign body clear spaces and the absence of asteroid bodies were more consistent with a granulomatous reaction to silicone than sarcoid. In previously published cases of cutaneous sarcoidosis occurring at injection sites, patients were younger, reported a history of scarring at sites of minor trauma, and clinically presented with red-brown papules that on histopathology was predominated by epithelioid granulomas without clear spaces [9–11]. Although serum angiotensin-converting enzyme (ACE) was not checked, it should be noted that an abnormal level would not have been diagnostic of sarcoidosis given the other likely diagnoses.

In addition to prior acupuncture, the patient reported a history of prior eyeliner tattooing, a minifacelift, and Botox. Though these are all potential sources of foreign body granulomas, the identified lesions did not correspond to the involved surgical sites. On histopathology, there was also no evidence of tattoo pigment nor birefringent suture material in any of the specimens as would be expected. Botox-related granulomas are unusual except in the context of sarcoidosis and would have lacked the numerous vacuolated clear spaces seen in our specimen. The patient denied prior facial filler, and the histopathological findings support her history. Commonly encountered fillers display characteristic histopathological findings that are used for identification [4]. Hyaluronic acid filler, for instance, would appear basophilic on hematoxylin-eosin stain. The identification of a clear exposure and material helped assuage concerns for underlying malignant or autoimmune processes and helped preclude further surgical intervention.

Treatment of silicone granulomas varies based on the size and location of the lesions. Small localized lesions are commonly excised but may require wide margins due to the risk of progressive scarring if silicone is allowed to migrate through layers of tissue. Disseminated lesions often require medical treatment. These include intralesional steroid injections and oral immunomodulators such as doxycycline, minocycline, celecoxib, allopurinol, and etanercept [12, 13]. Although patients respond well initially, recurrence is common after discontinuation of medical therapy and long-term immunosuppression may be required [14].

Acupuncture use in the United States is rising. According to the 2007 National Health Interview Survey that examined the use of complementary and alternative medicine, approximately 6.3% of the population or 14 million people have undergone a needling procedure [15]. Trigger points on the head, face, and neck are traditionally used to manage headaches, stress, and allergy symptoms [16]. Our patient only received acupuncture to the face. Lesions were found at jingming (Figure 1(a)), tongziliao (Figure 1(b)), taiyang (Figures 1(c) and 1(d)), bitong (Figure 1(e)), and quanliao (Figure 1(f)) which are targeted to alleviate eye strain, headache, stress, allergies, and facial pain, respectively.

Traditional needling practices are also being adapted for cosmetic purposes. Facial cosmetic acupuncture (FCA) has been touted to help with antiaging, skin rejuvenation, and restoration of muscle tone [17]. Although a small clinical trial in Korea has shown positive results based on objective changes on Moire facial topography and patient self-assessment, there is unfortunately an underrecognition of the potential side effects, including disfiguring silicone granulomas [14, 18]. This complication may be prevented with the use of silicone-free acupuncture needles, and early recognition by providers may prevent further disease.

## 4. Conclusions

Silicone granulomas may present decades after exposure and are important to consider on the differential for unexplained multifocal facial masses that follow an unnatural pattern. Diagnosis requires a thorough history, and in particular, providers must be aware of the potential for granuloma formation with the use of silicone-coated acupuncture needles.

## Data Availability

Data is available upon request through the corresponding author.

## References

[1] Winer L. H., Sternberg T. H., Lehman R., Ashley F. L. (1964). Tissue reactions to injected silicone liquids. *Archives of Dermatology*.

[2] Hu H. C., Fang H. W., Chiu Y. H. (2017). Delayed-onset edematous foreign body granulomas 40 years after augmentation rhinoplasty by silicone implant combined with liquid silicone injection. *Aesthetic Plastic Surgery*.

[3] Fallacara A., Manfredini S., Durini E., Vertuani S. (2017). Hyaluronic acid fillers in soft tissue regeneration. *Facial Plastic Surgery*.

[4] Requena L., Requena C., Christensen L., Zimmermann U. S., Kutzner H., Cerroni L. (2011). Adverse reactions to injectable soft tissue fillers. *Journal of the American Academy of Dermatology*.

[5] Yanagihara M., Fujii T., Wakamatu N., Ishizaki H., Takehara T., Nawate K. (2000). Silicone granuloma on the entry points of acupuncture, venepuncture and surgical needles. *Journal of Cutaneous Pathology*.

[6] Tschetter A. J. H. (2014). Silicone granulomas in the setting of acupuncture with silicone-coated needles. *Journal of Clinical and Investigative Dermatology*.

[7] Brito-Zeron P., Sellares J., Bosch X. (2016). Epidemiologic patterns of disease expression in sarcoidosis: age, gender and ethnicity-related differences. *Clinical and Experimental Rheumatology*.

[8] Noe M. H., Rosenbach M. (2017). Cutaneous sarcoidosis. *Current Opinion in Pulmonary Medicine*.

[9] Zargham H., O'Brien E. (2016). Cutaneous sarcoidosis at insulin injection sites. *Canadian Medical Association Journal*.

[10] Mermin D., Loustalan M. P., Doutre M. S. (2017). A case of hyaluronic acid injections triggering cutaneous sarcoidosis at previously treated sites. *Journal of the European Academy of Dermatology and Venereology*.

[11] Herbert V. G., Blödorn-Schlicht N., Böer-Auer A. (2015). Granulomatöse hautveränderungen an botulinumtoxin-A-injektionsstellen. *Der Hautarzt*.

[12] Beer K. (2009). Delayed onset nodules from liquid injectable silicone: report of a case, evaluation of associated histopathology and results of treatment with minocycline and celocoxib. *Journal of Drugs in Dermatology*.

[13] Chen T. A., Mercado C. L., Topping K. L., Erickson B. P., Cockerham K. P., Kossler A. L. (2018). Disseminated silicone granulomatosis in the face and orbit. *American Journal of Ophthalmology Case Reports*.

[14] Bashey S., Lee D. S., Kim G. (2015). Extensive facial sclerosing lipogranulomatosis as a complication of cosmetic acupuncture. *Dermatologic Surgery*.

[15] Barnes P. M., Bloom B., Nahin R. L. (2008). Complementary and alternative medicine use among adults and children: United States, 2007. *National Health Statistics Report*.

[16] White A., Cummings T. M., Cummings M., Filshie J. (2008). *An Introduction to Western Medical Acupuncture: Churchill Livingstone*.

[17] Barrett J. (2005). Acupuncture and facial rejuvenation. *Aesthetic Surgery Journal*.

[18] Yun Y., Kim S., Kim M., Kim K. S., Park J. S., Choi I. (2013). Effect of facial cosmetic acupuncture on facial elasticity: an open-label, single-arm pilot study. *Evidence-based Complementary and Alternative Medicine*.

